# The Pre-Transplant Drop in Panel-Reactive Antibodies Titer Evaluated Using Complement-Dependent Cytotoxicity (PRA-CDC) and the Risk of Early Acute Rejection in Sensitized Kidney Transplant Recipients

**DOI:** 10.3390/medicina54050066

**Published:** 2018-09-20

**Authors:** Aureliusz Kolonko, Beata Bzoma, Piotr Giza, Beata Styrc, Michał Sobolewski, Jerzy Chudek, Alicja Dębska-Ślizień, Andrzej Więcek

**Affiliations:** 1 Department of Nephrology, Transplantation and Internal Medicine, Medical University of Silesia, Francuska 20, 40-037 Katowice, Poland; piotr.giza@gmail.com (P.G.); beata.styrc@gmail.com (B.S.); awiecek@sum.edu.pl (M.S.); michalsobolewski911222@onet.pl (A.W.); 2 Department of Nephrology, Transplantation and Internal Medicine, Medical University of Gdańsk, Dębinki 7, 80-952 Gdańsk, Poland; bbzoma@gumed.edu.pl (B.B.); adeb@gumed.edu.pl (A.D.-Ś.); 3 Department of Internal Medicine and Oncological Chemotherapy, Medical University of Silesia, Reymonta 8, 40-027 Katowice, Poland; chj@poczta.fm

**Keywords:** acute rejection, immunization, panel-reactive antibodies, positive cross-match, waiting list

## Abstract

*Background*: The panel-reactive antibodies that use the complement-dependent cytotoxicity test (PRA-CDC) are still a standard method for monitoring the degree of immunization in kidney transplant candidates on active waiting lists in some countries, including Poland. The aim of this study was to analyze the relationship between the maximum and the last pre-transplant PRA titer on the percentage of positive cross-matches and rate of early acute rejection episodes. *Material and methods*: The retrospective analysis included 528 patients from two transplant centers. All patients were divided into three groups, depending on their peak and last pre-transplant PRA titers. There were 437 (82.8%) patients with peak PRA <20% (non-sensitized group, non-ST) and 91 (17.2%) patients with peak PRA >20%. Among the latter group, 38 had maintained PRA level >20% at the time of transplantation (sensitized patients, ST), whereas 53 had pre-transplant PRA ≤20% (previously sensitized patients, prev-ST). *Results*: The percentages of positive crossmatches were 76.9% in ST and 53.7% in prev-ST groups versus 18.4 in non-ST group (both *p* < 0.001). The acute rejection rates were 18.9, 17.6 and 6.8%, respectively (*p* < 0.001 for ST or prev-ST versus non-ST). The pre-transplant PRA titer drop did not decrease the risk of early acute rejection [OR = 1.09 (95% CI: 0.31–3.85)] in a multiple logistic regression analysis. The occurrences of primary graft non-function and delayed graft function were similar in all study groups. *Conclusions*: Previously immunized kidney transplant candidates even with substantial decrease in pre-transplant PRA-CDC levels are still at high immunological risk when compared with non-immunized patients, and they should receive lymphocyte-depleting induction therapy.

## 1. Background

The panel-reactive antibody (PRA) test is a routine screening measure to assess the degree of a potential kidney recipient’s sensitization, as a result of prior exposure to external HLA antigens during previous blood transfusions, pregnancies, or organ transplantations [1]. PRA titer is measured in all kidney transplant candidates allocated on an active waiting list, and its results, presented as peak (maximum historical) or the last pre-transplant PRA titers are taken into consideration at the moment of kidney transplantation for an optimal choice of immunosuppression protocol, including the type of induction therapy, if any. To date, it was demonstrated that the incidence of positive cross-matches increase in patients with peak PRA activity 1–50% and >50% [2]. Similarly, the incidence of acute rejection episodes was also greater in patients with peak PRA >0% [3,4].

Despite enormous progress in the field of anti-HLA antibody detection and the assessment of the potential kidney transplant candidate’s degree of sensitization, the number of countries have not introduced the universal single bed antigen detection among waiting list patients, mostly due to the shortage of financial resources [5,6]. In some countries, including Poland, PRA titer is measured using the complement-dependent cytotoxicity test (PRA-CDC) as a standard procedure [6]. In our center, patients with PRA <20% are arbitrarily classified as subjects with low immunological risk. Based on our clinical observations we hypothesized that in historically highly sensitized patients, the low last pre-transplant PRA titer might not reflect the real degree of sensitization. Thus, we have decided to use the percentage of positive cross-matches in a given patient (out of all crossmatches performed during the stay on an active waitlist) as a surrogate marker of sensitization. Additionally, we have analyzed different aspects of early kidney graft function, with detailed types of acute rejection episodes.

The aim of this study was to analyze the effect of the peak and the last pre-transplant PRA titers on the percentage of positive crossmatches, delayed graft functions (DGF), primary graft non-function (PGN), and early acute rejection (AR) episodes. In order to increase the statistical strength, we have performed our analysis based on patients transplanted in two Polish transplant centers.

## 2. Materials and Methods

This retrospective analysis included 528 out of 1078 consecutive kidney transplant recipients (KTRs): 327 in the Silesian (transplanted in years 2012–2017) and 201 in the Pomeranian (transplanted in years 2012–2015) centers in Poland who met the inclusion criterion; i.e., more than 5 completed (positive or negative) crossmatches performed via the countrywide kidney transplant matching system before successful transplantation, including the last one at the day of transplant. The final analysis was carried out in March 2018. In Poland, during each donor-recipient matching procedure the recipients are finally chosen out of the list of patients with a negative crossmatch, based on the higher number of transplant points. We have excluded living donor transplants, simultaneous pancreas-kidney recipients and those KTRs who have been classified and transplanted in an urgent mode due to a lack of dialysis vascular access. No patient was transplanted across the positive crossmatch. 

This investigation was carried out following the rules of the Declaration of Helsinki. The Bioethics Committee of the Medical University of Silesia granted the permission for this study, informed consent was not deemed necessary as the study does not fulfill the criteria of a medical experiment (KNW/0022/KB/284/15). All data were analyzed anonymously based on the prospectively maintained databases of both transplant centers.

All patients were divided into three groups, depending on their maximum and last pre-transplant PRA titers. The majority of the patients had maximal PRA titer <20% and were allocated to the non-sensitized group (non-ST). Patients with both maximal and the last pre-transplant PRA >20% formed the currently sensitized group (ST). Patients with maximum PRA titer >20%, but a pre-transplant PRA <20%, were characterized as a previously sensitized group (prev-ST).

The immunosuppressive protocol data and early kidney graft outcomes were reported by both transplant centers. All patients received a triple immunosuppressive regimen, based mostly on tacrolimus, mycophenolate mofetil or sodium, and steroids. In a whole study group, 45.1% of patients were treated with induction therapy, using basiliximab (27.3%) or antithymocyte globulin (18.1%).

Early kidney graft function was assessed including PGN, DGF, and AR episodes. DGF was defined as a need of dialysis therapy within the first post-transplant week. Early AR during the first post-transplant hospital stay [mean 21 (95% CI: 20–22) days] was defined based on a clinical diagnosis, confirmed by kidney biopsy. The type of AR (T-cellular, vascular, mixed, or humoral AR) was also analyzed.

For the statistical analyses, the 12.5 version of Statistica software (StatSoft, Cracow, Poland) was used. Distribution of the examined variables was checked by the Shapiro–Wilk test. Data was presented as mean values and 95% confidence interval. Categorical variables were compared using ANOVA and χ^2^ tests for trend. Correlation coefficients for PRA titers were calculated according to Spearman due to their nonparametric distribution. In the combined subgroup of prev-ST and ST patients we performed logistic regression analysis for the occurrence of acute rejection episodes as a dependent variable, with potential explanatory variables selected on the basis of univariate analysis (recipient gender, retransplant, HLA class II mismatch at least in 1 *locus*, no induction therapy and the pretransplant PRA drop). The threshold for statistical significance was set at 0.05.

## 3. Results

There were 437 (82.8%) patients with peak PRA <20% (non-ST) and 91 (17.2%) patients with peak PRA >20%. Among the latter group, 38 patients had maintained PRA level >20% at the time of transplantation (sensitized patients, ST), whereas 53 patients had pre-transplant PRA ≤20% (previously sensitized patients, prev-ST). The clinical characteristics of three study subgroups are given in Table 1.

There was a significant difference in the time of dialysis therapy before transplantation, however, the dialysis vintage of patients from ST and prev-ST subgroups was similar (Table 1). Also, the percentage of re-transplants was significantly higher among ST and prev-ST groups. The ST patients had a significantly greater HLA class II mismatch, as their transplant points on the last negative cross-match (at the day of kidney transplant) came partly as a consequence of their higher PRA sensitization degree, according to the standard cross-matching procedure. Of note, the prev-ST patients spent the longest time on the active waiting list (Table 1).

The percentage of positive cross-matches among all such procedures performed during the time on an active waiting list was 76.9% in ST group versus 18.4% in non-ST group (*p* < 0.001). Of importance, in prev-ST patients the percentage of positive cross-matches was also significantly greater (53.7%) than in the non-ST group. There was a stronger correlation between the percentage of positive cross-matches and peak PRA (R = 0.487, *p* < 0.001) than between the percentage of positive cross-matches and last pre-transplant PRA (R = 0.401, *p* < 0.001).

**Figure 1:**
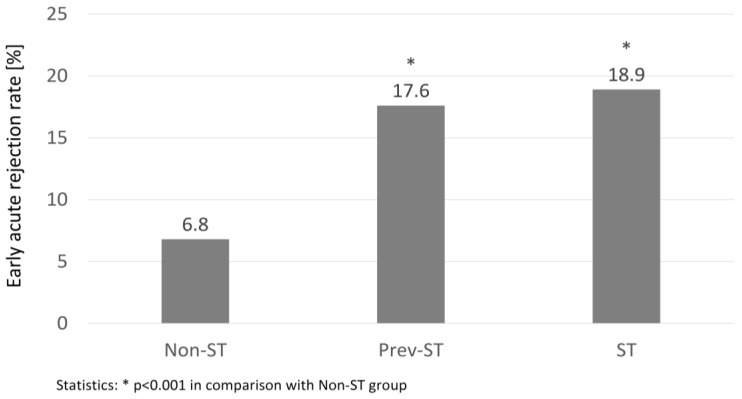
The percentage of early acute rejection episodes in the groups of non-sensitized (Non-ST), previously sensitized (Prev-ST), and sensitized (ST) kidney transplant recipients.

The overall incidence of DGF, PGN, and AR in the whole study group was 28.0, 1.7, and 8.5%, respectively. There were no significant differences in PGN and DGF occurrence in all study groups. Contrary, the rates of AR episodes in the early post-transplant period were significantly higher in both prev-ST and ST groups in comparison to the non-ST group (Figure 1). Moreover, the study subgroups differed in regards to the structure of the AR subtypes: the percentage of cellular or mixed rejection was highest in non-ST group (51.7 vs. 44.4 and 14.3% in prev-ST, NS and ST, *p* < 0.05, respectively), whereas humoral rejection episodes were the most common type in the ST group (57.1 vs. 33.3 and 17.2% in prev-ST and non-ST, respectively, both NS). The percentages of vascular rejection were similar in all groups. In addition, the percentage of positive cross-matches was significantly higher in patients with AR than those without AR (37 vs. 25%, *p* = 0.003).

The multiple logistic regression analysis performed in the combined subgroup of prev-ST and ST patients revealed that the pre-transplant PRA titer drop does not decrease the risk of an early acute rejection episode. In this analysis, only HLA class II mismatch was significantly associated with the increased risk of AR (Table 2).

## 4. Discussion

In this study we have demonstrated that previously sensitized kidney transplant candidates with last pre-transplant PRA-CDC level <20% still present a substantial level of sensitization. It resulted in a large percentage of positive crossmatches during waitlisting, and in the frequent occurrence of early AR as compared to never-sensitized patients. In previously sensitized patients, in fact, the frequency of AR was quite similar to patients with persistently positive PRA titers, whereas the percentage of positive crossmatches was much closer to the latter group than to the non-sensitized group. Importantly, as high historical but low current PRA titers were usually recognized as low-immunological risk at the time of transplantation, those patients have often received less powerful induction regimen, with only 47.2% treated with a polyclonal antibody in the study group.

When analyzing the kidney graft survival, it was clearly demonstrated that it is markedly affected by the increased pre-transplant PRA titer [7,8,9]. Moreover, some authors showed that patients with historically high, but currently low PRA, had a graft survival similar to those who had moderately increased PRA titer in their pre-transplant serum samples [8,9]. Even earlier, Sanfilippo et al. doubted the prognostic value of the peak PRA titer for graft outcome in patients with a substantial decrease of PRA titer being on the active waiting list [10]. In addition, they have suggested that a substantial decrease in PRA titer prior the transplantation does not necessarily indicate a decrease in potential recipient alloreactivity [11]. However, they classified “large” a PRA drop based on absolute PRA values as a change of < or ≥40 percentage points, and analyzed only irreversible graft rejections over the period of two years. In our present study, we have demonstrated for the first time that the peak PRA titer, but not the last pre-transplant one, is associated with increased incidence of AR in the early post-transplant period and substantially higher percentage of positive cross-matches while being on an active waiting list. It may suggest the persisted ability of immunological memory cells to induce an accelerated instead of primary immune response, despite a substantial PRA titer decrease prior to transplantation [9,12]. As a consequence, the total waiting time was also significantly longer in prev-ST, as compared with the non-ST group. Of note, it was demonstrated that a peak PRA titer of >20% significantly diminishes the probability of transplantation of the kidney received from the deceased donor [13]. Our observation concerning the increased percentage of positive cross-matches in KTRs both with a previous or current PRA level of >20% may explain this finding.

The most important conclusion of our work is that potential kidney transplant recipients with high historical, but a current PRA titer of <20%, are more prone to receive non-optimal induction therapy with basiliximab or even no induction, if the results of current solid-phase cross-match assays are not available. Such a non-optimal choice of induction might be further caused by the most often used PRA titer thresholds for highly-sensitized patients (>50 or >80%). As a consequence, a long-term graft survival may be worse in these patients [14]. 

Nevertheless, even in patients with known anti-HLA pre-transplant status, PRA titer remains essential information, determining the clinical decisions. On the other hand, based on single antigen bead testing, a so called virtual panel reactivity (vPRA) value is calculated that reflects the percentage of organ donors that are not eligible for the patient [15]. Virtual crossmatch based on vPRA results gives the chance to predict the outcome of a biological crossmatch in the early stages of typing and may shorten cold ischemia time, thus improving the results of transplantation [16].

Our study was limited by the lack of routine protocol biopsies in the analyzed cohort of patients. Secondly, there were some differences in immunosuppressive regimens between these two transplant centers. Lastly, PRA–CDC tests were performed according to the same protocol in two laboratories (one per center), however no comparison between these laboratories was done.

## 5. Conclusions

In summary, kidney transplant candidates with a substantial decrease in pre-transplant PRA-CDC level during waitlisting are still at high immunological risk as compared with non-sensitized patients, and should receive lymphocyte-depleting induction therapy. The peak (historical) PRA titer is a valuable marker of sensitization in the lack of solid-phase cross-match assays before transplantation.

## Figures and Tables

**Table 1:** Characteristics of the three study groups, defined on the maximum and last pre-transplant PRA titers (Non-ST group: max PRA ≤20%; Prev-ST group: max PRA >20% but last pre-transplant PRA ≤20%; ST group: max PRA and last PRA >20%).

	Group 1	Group 2	Group 3	ANOVA/Chi^2^
	Non-ST	Prev-ST	ST	
	N = 437	N = 53	N = 38	
Age [yrs]	50.6 (49.3–51.8)	51.5 (48.0–55.0)	47.9 (43.7–52.1)	0.40
Gender (M/F)	283/154	24/29	15/23	<0.001 *
Dialysis vintage [mo]	42 (39–45)	65 (54–76)	69 (52–86)	<0.001 for 2. and 3. vs. 1.
Retransplant [%]	11.5	37.7	55.3	<0.001 *
HLA mismatch I	2.2 (2.1–2.3)	1.9 (1.6–2.2)	2.0 (1.7–2.3)	0.16
HLA mismatch II	0.5 (0.5–0.6)	0.6 (0.4–0.8)	0.8 (0.5–1.0)	0.02
PRA last [%]	0.4 (0.2–0.5)	3.9 (2.3–5.5)	48.6 (41.2–55.9)	<0.001 (0.01 for 2. vs. 1.)
PRA max [%]	1.7 (1.3–2.1)	38.4 (33.2–43.6)	61.5 (53.3–69.7)	<0.001 *
CyA/Tc [n]	83/351	4/49	5/33	0.09
MMF [%]	97.7	100	100	0.34
Induction total n (%)	155 (35.8)	45 (84.9)	38 (100)	<0.001 *
SIMU n (%)	118 (27.2)	20 (37.7)	5 (13.2)	Chi^2^ test 0.03
ATG n (%)	37 (8.5)	25 (47.2)	33 (86.8)	<0.001 *
XM+ [%]	18.4 (16.7–20.1)	53.7 (46.6–60.7)	76.9 (70.6–83.2)	<0.001 *
Time on the waitlist [mo]	9.6 (8.7–10.4)	14.8 (12.0–17.6)	12.5 (8.9–16.0)	0.01 for 2. vs. 1.
DGF [%]	29.4	39.2	32.4	0.39
PGN [%]	1.6	1.9	2.6	0.89

Data shown as means and 95% CI or frequencies. HLA: human leukocyte antigen; PRA: panel-reactive antibodies; CyA: cyclosporine. Tc: tacrolimus; MMF: mycophenolate mofetil or mycophenolate acid; SIMU: Simulect; ATG: antithymocyte globulin; XM+: the percentage of positive crossmatches among all crossmatches performed; DGF: delayed graft function; PGN: primary graft non-function; AR: acute rejection. Statistics: * For trend. HLA class I denotes 2 A *loci* and 2 B *loci*. HLA class II denotes 2 DR *loci*.

**Table 2:** Multiple logistic regression analysis performed in the subgroup of prev-ST and ST patients (n = 91) for the risk of early acute rejection.

Independent Variable	Beta	SE	OR	95% CI	*p* Value
Recipient gender (M vs. F)	−0.97	0.65	0.38	0.11–1.37	0.14
Retransplant	0.26	0.60	1.30	0.40–4.26	0.67
Lack of induction therapy	1.23	1.03	3.43	0.46–25.8	0.23
Any HLA class II mismatch	1.49	0.75	4.43	1.02–19.3	<0.05
PRA titer drop	0.09	0.64	1.09	0.31–3.85	0.89
